# Rapid resolution of widespread cutaneous lichen planus and generalized pruritus in an elderly patient following treatment with dupilumab

**DOI:** 10.1016/j.jdcr.2022.10.019

**Published:** 2022-10-29

**Authors:** Soroush Kazemi, Morgan Murphrey, Jason E. Hawkes

**Affiliations:** Department of Dermatology, University of California Davis, Sacramento, California

**Keywords:** dupilumab, IL-4, IL-13, lichen planus, pruritus, Th1, Th2, IFN, interferon, LP, lichen planus, NRSi, numeric rating scale itch intensity, TSLP, thymic stromal lymphopoietin

## Introduction

Lichen planus (LP) is a rare inflammatory disease that affects <1% of the population and is most common in middle-aged adults.^1^ Classically manifesting as violaceous, polygonal, flat-topped, and pruritic papules or plaques on the skin, LP has a pleiomorphic presentation and may involve the hair, nails, or mucosal tissues.^2^ Pruritus is a common symptom of LP and several disease associations have been described including trauma, medications, hepatitis infection, and mucosal exposure to dental restorative materials.^2^ While the exact pathophysiology of LP is not fully understood, cytotoxic (CD8^+^) T lymphocytes directed against the affected tissue represent a common feature shared among LP variants.^3^^,^^4^ No targeted therapies are approved for the treatment of LP and current treatment strategies include the use of broad-acting immunomodulatory agents such as corticosteroids, retinoids, azathioprine, mycophenolate mofetil, and methotrexate.^1^

Dupilumab is a monoclonal antibody that blocks the IL-4Rα subunit, thereby modulating the Th2 immune response via dual inhibition of IL-4 and IL-13.^5^ Dupilumab is Food and Drug Administration-approved for treatment of moderate-to-severe atopic dermatitis, asthma, chronic rhinosinusitis with nasal polyps, prurigo nodularis, and eosinophilic esophagitis. It has also shown tremendous promise as a treatment for non-atopic and pruritus-related conditions (eg, keloids and bullous pemphigoid).^6^ The role of dupilumab in the management of LP and LP-associated pruritus has not been studied in clinical trials. Here, we describe an elderly patient with widespread cutaneous LP and severe, generalized pruritus who experienced rapid resolution of her skin lesions and itching following treatment with dupilumab.

## Case report

A 92-year-old female with a history of type II diabetes and hypertension was referred to dermatology for the evaluation and treatment of a widespread rash and generalized pruritus lasting more than 2 years. High potency topical steroids were largely ineffective. Her itch symptoms were unresponsive to oral anti-histamines and significantly interfered with her sleep. HIV and hepatitis screening tests were negative and discontinuation of her oral medications for more than 6 weeks did not result in improvement of her skin or itching. Prior treatment with oral prednisone provided temporary improvement, but disease recurrence was noted upon discontinuation. Patient screening for malignancy, including blood work and computed tomography imaging of the chest and abdomen, was normal and completed prior to the dermatology consultation.

**Fig 1:**
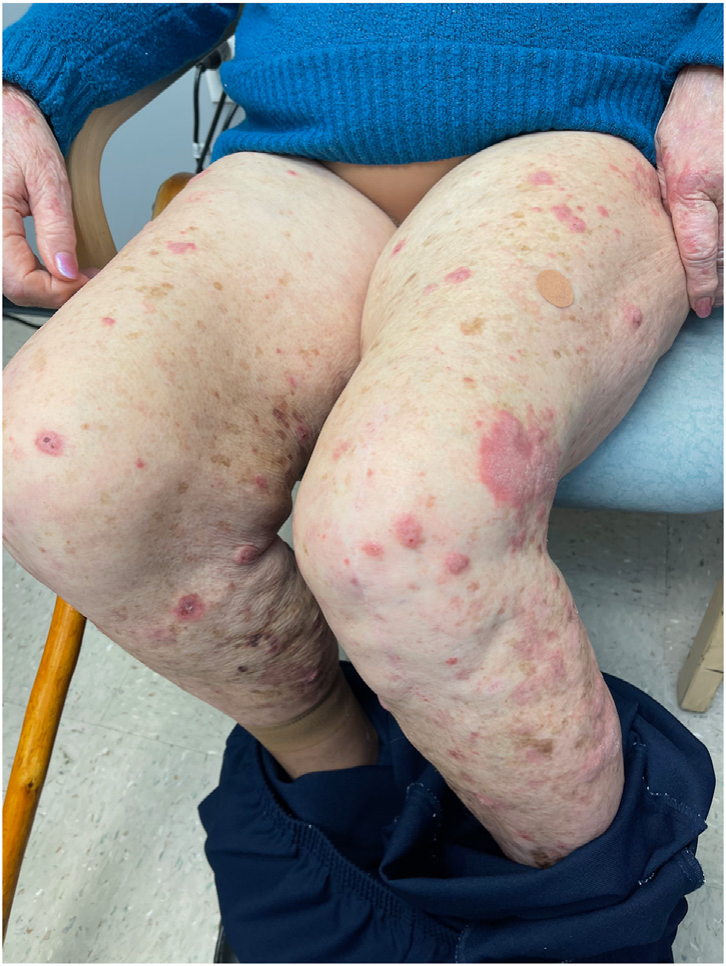
A clinical photo of lower extremities prior to the initiation of dupilumab treatment.

**Fig 2:**
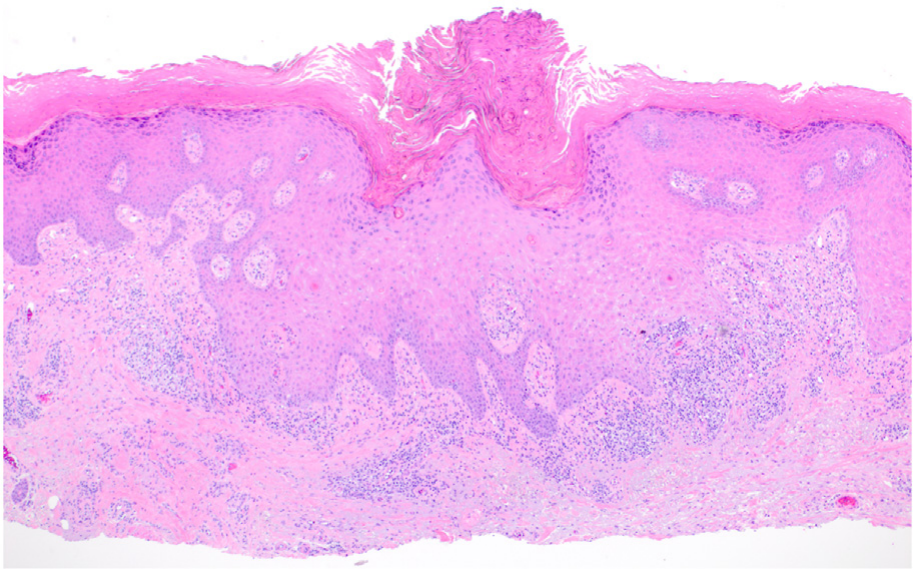
Representative low-magnification hematoxylin-eosin stain of a skin biopsy specimen taken from the left lateral knee (original magnification ×100).

**Fig 3:**
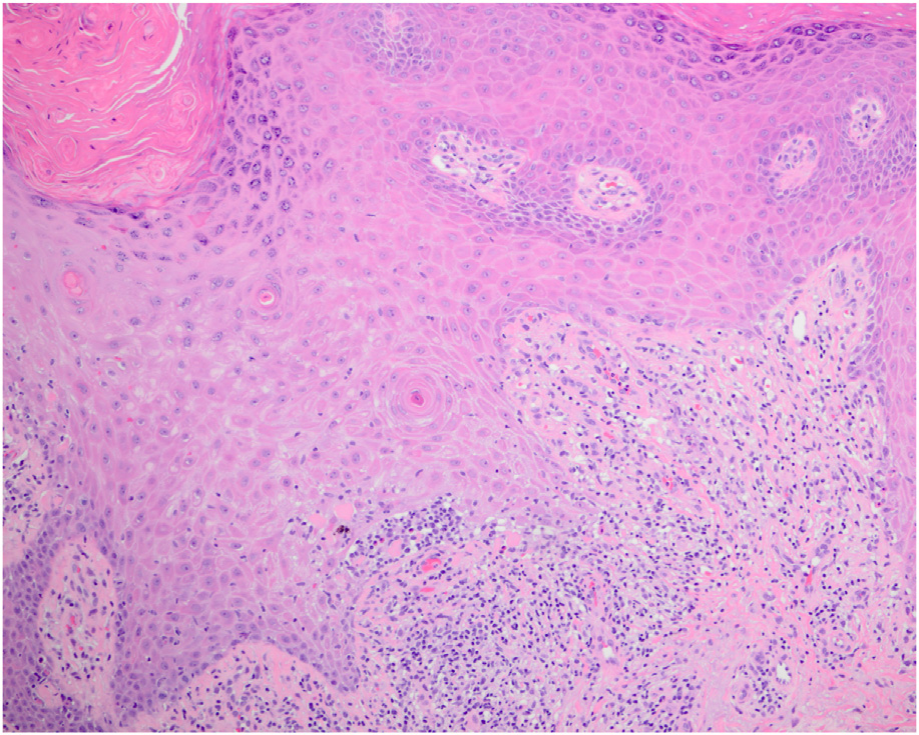
Representative high-magnification hematoxylin-eosin stain of a skin biopsy specimen taken from the left lateral knee (original magnification ×100).

**Fig 4:**
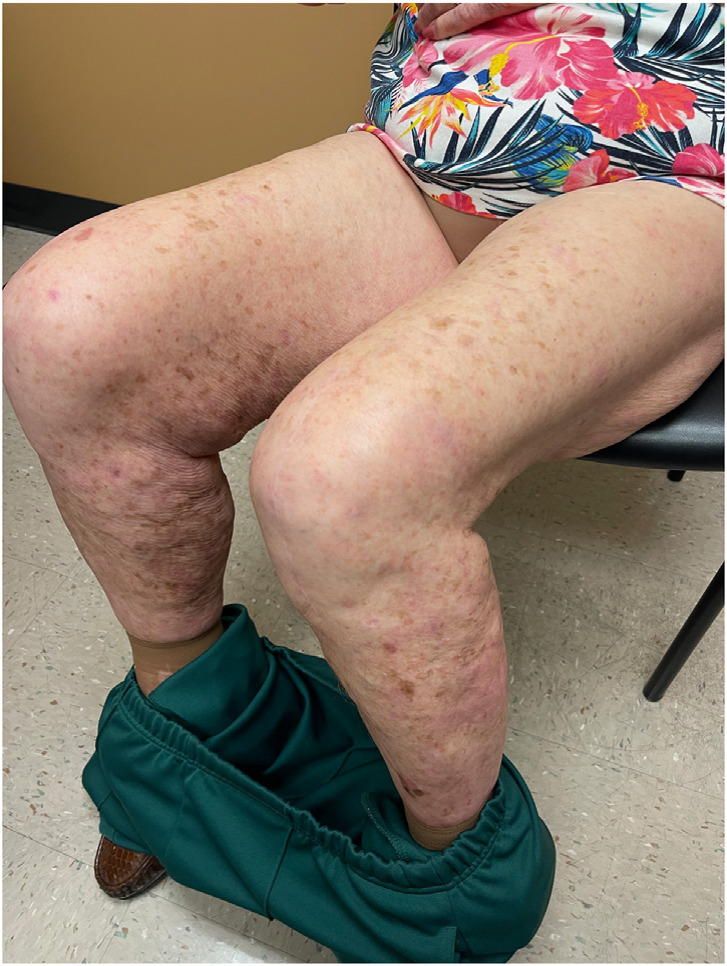
A clinical photo of lower extremities following 2 months of dupilumab treatment.

On exam, we observed widespread violaceous, flat-topped papules and plaques on the trunk, and all 4 extremities with scattered excoriations (Fig 1). No associated nail, hair, or mucosal abnormalities were noted. Skin biopsies of the left lateral knee and upper chest were performed and showed orthoparakeratosis, acanthosis with hypergranulosis, spongiosis, sparse eosinophils, and a band-like lymphohistiocytic infiltrate that focally obscured the dermal-epidermal junction consistent with a diagnosis of cutaneous LP (Figs 2 and 3). Importantly, the patient reported worsening and enlargement of the lesion on her left lateral knee following the skin biopsy (ie, isomorphic response), which is visible as a depressed macule within a larger plaque (Fig 1). Dupilumab was offered as a treatment option given the patient’s advanced age, inability to take methotrexate or cyclosporine due to her chronic kidney disease, and potential drug interactions with her diabetes and blood pressure medications. After initiation of dupilumab (600 mg initially followed by 300 mg every 2 weeks), the patient reported rapid resolution of her itching within 3 weeks and complete skin clearance after 2 months (Fig 4). Her skin remains clear with no reported adverse events related to ongoing dupilumab therapy.

## Discussion

Although LP is generally recognized as a T-cell mediated inflammatory condition, the specific immune cell populations and the pathogenic cytokines driving the development of this disease have not been fully elucidated. Several studies found elevated CD4^+^ and CD8^+^ cell populations in the skin and blood of LP patients along with a strong Th1 or interferon (IFN)-γ signaling profile.^3^^,^^4^ *IFNG*, *IL4*, *IL12A*, *IL21*, and *TNF* are the most commonly expressed genes found to be increased in cutaneous LP tissues.^3^ However, other studies suggest a mixed T lymphocyte immune response, including Th2 cell populations producing increased amounts of IL-4, IL-13, and IL-10 cytokines.^3^^,^^4^ The potential role of Th2 signaling in the development of LP is further supported by increased levels of thymic stromal lymphopoietin^7^ and IL-25^8^ in oral LP specimens, both of which act as alarmins by promoting a Th2-based immune response and pruritus. These findings suggest a potential pathogenetic role of activated Th2 lymphocytes and their respective cytokines (eg, IL-4 and IL-13) in the immunopathogenesis of LP and its associated pruritus.

To date, there are no Food and Drug Administration-approved therapies available for the treatment of LP. Dupilumab has been clinically proven as a highly effective inhibitor of Th2 inflammation and anti-pruritic agent in multiple tissues across various disease states.^9^ Several studies have explored the potential therapeutic benefit of dupilumab for the treatment of chronic pruritus, regardless of the specific underlying etiology. A recent case series evaluating 20 patients with recalcitrant pruritus (including a non-elderly patient with cutaneous LP) treated with dupilumab reported reduced itching in all study participants and complete itch resolution in 60% of patients with an average Numeric Rating Scale Itch Intensity reduction of 7.55.^10^ We observed a similar reduction in the generalized, severe itch of our LP patient (Numeric Rating Scale Itch Intensity of 0) as well as complete resolution of her skin lesions following dupilumab therapy, thereby highlighting the dual benefit of this selective biologic for the blockade of type 2 inflammation and pruritus in cutaneous LP. It also underscores the potential safety and clinical utility of dupilumab for the treatment of chronic LP in elderly patients who have an increased risk of adverse events with traditional systemic immunosuppressants due to age-related physiologic changes, polypharmacy, and concomitant disease comorbidities.

In summary, LP is a heterogeneous, pruritic, inflammatory condition with a complex immunopathogenesis involving cytotoxic and mixed (Th1 and Th2) helper T lymphocyte populations. Recent human translational studies provide strong evidence for the role of type 2 inflammation in the pathogenesis of LP and serve as the impetus to further investigate the efficacy of dupilumab as a future treatment for this condition. The effectiveness of dupilumab for the treatment of all LP clinical variants is unknown and warrants further investigation in dedicated clinical trials where larger patient cohorts can be studied.

## Conflicts of interest

None disclosed.
